# A novel electromagnetic guidance ultrasound system on radial artery cannulation: a prospective randomized controlled trial

**DOI:** 10.1186/s12871-021-01244-6

**Published:** 2021-01-18

**Authors:** Namo Kim, Hyun Il Kim, Do-Hyeong Kim, Dahee Park, Sei Han Song, Hyo-Jin Byon

**Affiliations:** 1 Department of Anesthesiology and Pain Medicine, Anesthesia and Pain Research Institute, Yonsei University College of Medicine, 50-1 Yonsei-ro, Seodaemun-gu, Seoul, 03722 Republic of Korea; 2 Department of Anesthesiology, The Armed Forces Yangju Hospital, Yangju, Republic of Korea; 3 Department of Anesthesiology and Pain Medicine, Gangnam Severance Hospital, Anesthesia and Pain Research Institute, Yonsei University College of Medicine, Seoul, Republic of Korea

**Keywords:** Electromagnetic fields, Ultrasonography, Radial artery, Cannulation, Catheter

## Abstract

**Background:**

Radial artery cannulation can cause complications such as haematoma formation or thrombosis due to its small diameter. Recently, a novel ultrasound device equipped with an electromagnetic guidance system was introduced, showing the path and alignment of the needle during the procedure. The aim of this study was to investigate the effects of this novel system on both success and complication rates during radial artery cannulation under ultrasound guidance.

**Methods:**

In this randomized controlled trial, 76 adults scheduled for neurosurgery requiring radial artery cannulation were recruited. In group E (*n* = 38), radial artery cannulation was performed using the electromagnetic guidance ultrasound system, whereas in group C (n = 38), the procedure was performed using conventional ultrasound guidance. The success rates of cannulation on the first attempt, cannulation times, number of attempts, and incidence of complications were compared between the two groups.

**Results:**

There was a significant difference in the success rates on the first attempt between the two groups (group C = 78.9% vs. group E = 94.7%, *P* = 0.042). Incidences of posterior wall puncture and haematoma formation (group C = 8 vs. group E = 1; *P* = 0.028) were significantly lower in group E than in group C. The median cannulation time for successful attempts was comparable between groups.

**Conclusions:**

Use of the novel electromagnetic guidance system resulted in a better success rate on the first attempt and a lower incidence of complications during radial artery cannulation.

**Trial registration:**

This study was registered at http://cris.nih.go.kr (registration number: KCT0002476).

## Introduction

Arterial cannulation is a common and important procedure for critically ill patients whose blood pressure must be measured directly and continuously, or when blood samples for laboratory testing must be obtained frequently during surgery or in the intensive care unit [1]. The use of dynamic hemodynamic monitors instead of traditional static hemodynamic monitors has increased recently to predict fluid responsiveness. Some fluctuating hemodynamic parameters like pulse pressure variation or systolic pressure variation are determined by analysing arterial waveforms obtained from an arterial catheter [2].

However, there are some disadvantages when using arteries for catheterization. Arteries generally have relatively small diameters and are prone to rupture by the high blood pressure delivered by the heart [3]. When a small artery is chosen for catheterization or the patient is obese, arterial palpitation is difficult. In such cases, arterial catheterization can fail or cause complications such as thrombosis, haematoma, embolization, arteriovenous fistula, and limb ischaemia [4–6]. Ultrasound guidance has proven useful for guiding arterial catheterization. Using an ultrasound device, a physician can identify an artery for catheterization, monitor the needle entering the artery, and confirm the placement of the catheter in the artery [6, 7]. However, ultrasound guidance doesn’t always guarantee the success of arterial cannulation or prevent complications. In one study, ultrasound guidance failed to prevent puncture of the vascular posterior wall in a mannequin despite good median confidence by the healthcare provider regarding the appropriate needle placement [8]. Additionally, the success rate of cannulation under ultrasound guidance is affected by the provider’s skill and experience [9].

Recently, the eZono™ 4000 (eZono AG, Jena, Germany) portable ultrasound device, which is equipped with an electromagnetic guidance system (eZGuide™), was introduced, showing the path and alignment of the needle relative to the probe during the procedure [10]. It also shows the expected route and tip of the needle. In several studies using a gel phantom model, the novel electromagnetic guidance system appears capable of reducing the incidence of complications and improving the success rate of invasive procedures among providers with or without sufficient experience with radial artery cannulation under ultrasound guidance [11–13]. However, no clinical in vivo studies have been conducted on the efficacy of the electromagnetic ultrasound guidance system during invasive procedures such as central venous catheterization or radial artery cannulation. The aim of this study was to compare the success and complication rates during arterial cannulation with or without this novel electromagnetic guidance system.

## Methods

This study was approved by the Institutional Review Board of Severance Hospital, Yonsei University Health System (approval number: 4–2016-1041) and was registered at http://cris.nih.go.kr (registration number: KCT0002476, date of first registration: 03/04/2017). The study had been carried out in accordance with the Declaration of Helsinki of the World Medical Association revised in 2013 for experiments involving humans. Written informed consent was obtained from all patients. Patients who required clinically indicated arterial cannulation from March 3rd 2017 to November 16th 2017 were enrolled in the study. Exclusion criteria included patients who had vascular malformations, coagulation abnormalities, or peripheral arterial occlusive disease, or who were hemodynamically unstable, or had undergone repeat or emergency surgeries. The participants were randomly allocated into two groups using a computerized, randomized table: the eZono group under electromagnetic ultrasound guidance (group E, *n* = 38) and the control group under conventional ultrasound guidance (group C, *n* = 38). The allocations were concealed in sequentially numbered, sealed, opaque envelopes.

After the placement of routine monitors, general anaesthesia was induced. Patients were intubated using a tracheal tube and mechanically ventilated. Patients were placed in the supine position, and an anaesthesiologist performed an Allen test to confirm whether blood circulation of the hand was normal. The wrist was extended over a roll of sheet, and the hands of the patients were positioned in dorsiflexion at 45 °. A 20-G, 1.1 × 30-mm Angiocath Plus™ catheter (Becton Dickinson Infusion Therapy Systems Inc., Sandy, Utah, USA) was used; before puncturing the skin, the provider applied the ultrasound gel and examined the anatomical structure of the tissue around the radial artery using the ultrasound view. Arterial cannulation was performed by a single anaesthesiologist who had successfully performed arterial cannulation under electromagnetic ultrasound guidance more than 50 times.

**Fig. 1:**
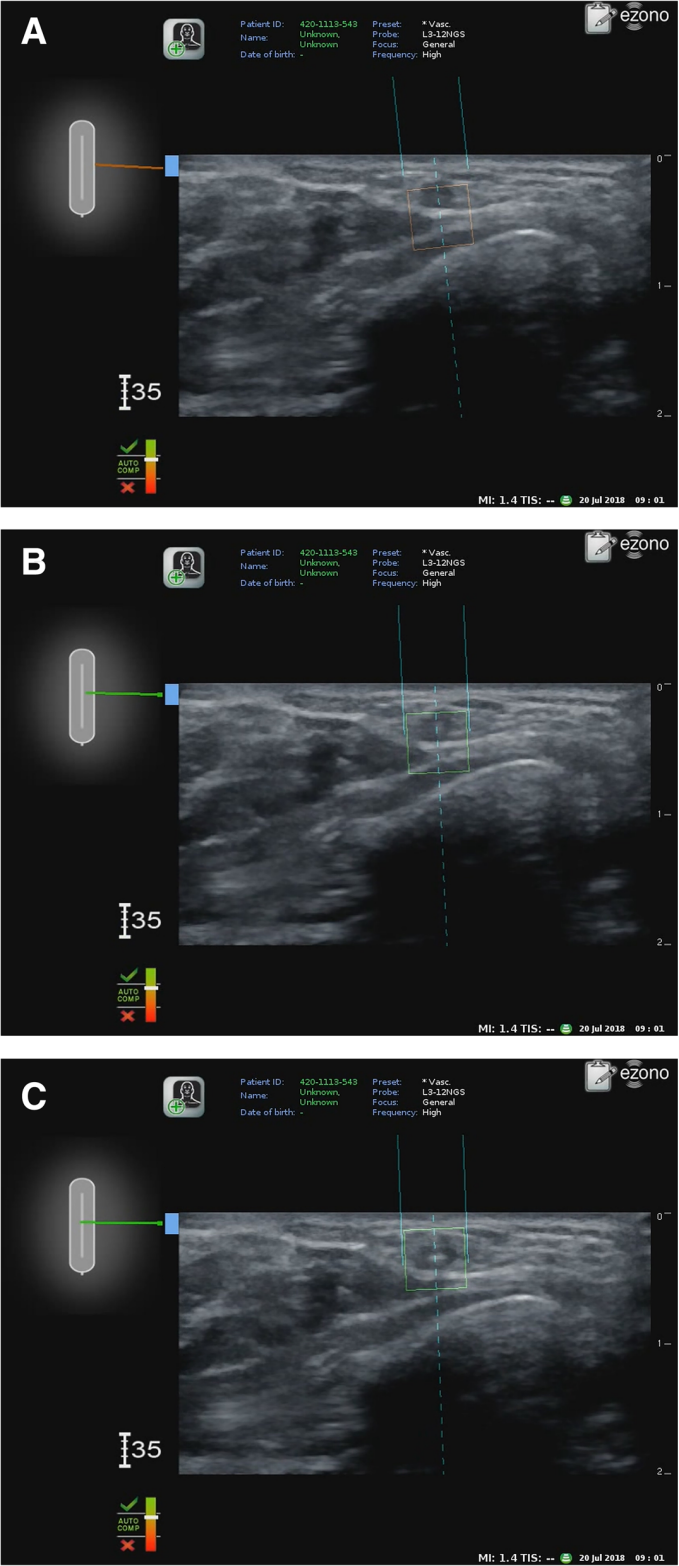
**a**. Radial artery cannulation using electromagnetic guidance system — Before alignment. The screen shows the actual position (two solid lines), expected route (dotted line), and tip of the needle (the indicator box). **b**. After alignment. The indicator box changed from red to green after the alignment of the needle. **c**. After puncture. The actual tip of the needle appeared in the middle of the indicator box

In group E, the electromagnetic guidance system (eZono 4000, eZono AG, Jena, Germany) was turned on, and a linear probe (3–12 MHz, NGS linear transducer, eZono AG) was placed perpendicular to the radial artery to obtain a short-axis view. After magnetizing the needle, the provider punctured the skin and inserted the needle perpendicular to the long axis of the probe—the out-of-plane approach—while maintaining an optimal view on the screen. The screen showed the expected route of the needle as a dotted line, the actual position of the needle as two solid lines, and the expected tip of the needle as a square box (the indicator box). The location and direction of the needle relative to the probe were shown in the upper left screen, and the needle changed in appearance on the screen from red to green when its tip reached the plane of the long axis of the probe (Fig. 1). The provider then advanced the needle toward the radial artery until the needle in the upper left screen appeared green while adjusting the needle direction to maintain the dotted line and the square indicator box targeting the radial artery in the correct orientation on the screen. The puncture of the radial artery by the needle was confirmed by the occult blood that appeared in the needle. In group C, radial artery cannulation was performed using the conventional technique under ultrasound guidance [7], which is the same technique as that used in group E except that no electromagnetic guidance was used during the procedure. If the provider failed to puncture the radial artery, the needle was removed, and the procedure was attempted again.

Both heart rate and blood pressure were recorded during the arterial cannulation. The transverse and longitudinal diameters of the radial artery, and the depth from the skin to the artery were recorded on the ultrasound view. The puncture site, cannulation time, number of attempts, and complications (posterior wall puncture, haematoma formation, and thrombosis formation) were recorded during the procedure. The cannulation time was defined as the time taken from the initial skin puncture to the successful insertion of the arterial catheter. The number of attempts was defined as the number of new skin punctures required to puncture the radial artery. A posterior wall puncture was defined when withdrawal of blood in the needle stopped after confirming the presence of occult blood while advancing the needle, or when pulsatile blood appeared during the withdrawal of the needle. A haematoma was defined as a solid swelling of clotted blood around the artery confirmed by ultrasound, and a thrombosis was defined as a local coagulation or clotting of blood in the artery confirmed by ultrasound.

Sample size estimation was performed in accordance with the results of a previous study reporting a 62% of success rate on the first attempt of arterial cannulation under ultrasound guidance [7]. We estimated that a random assignment of 76 subjects was required to detect a difference of 30% in success rate on the first attempt, with 80% power at the 5% significance level, which takes into consideration the usual 10% loss of study participants. Statistical analyses were performed using IBM SPSS Statistics for Windows, version 25.0 (IBM Corp., Armonk, N.Y., USA). Normality tests were performed using the Kolmogorov-Smirnov test. All data are expressed as the mean (SD or range), number (%), or median [interquartile range (IQR)] as indicated. Data between groups were compared using the χ^2^ test, the Mann-Whitney U-test, and the Student’s *t* test as appropriate. Statistical significance was defined as a *P* value less than 0.05.

## Results

A total of 76 adult patients were included in this study, and all radial artery cannulations were successfully performed. No significant differences were observed in the patient demographic data, such as age, height, weight, and sex, between the two groups (Table 1). Heart rate, and systolic and diastolic blood pressures measured during arterial cannulation did not differ between the two groups. No differences in the transverse and longitudinal diameters of the radial artery, or arterial depth at the puncture site between the two groups were observed (Table 2).

**Table 1:** Patient demographic data and vital signs during arterial cannulation

Parameter	Group C (*n* = 38)	Group E (*n* = 38)	*P* value
Age (years)	52.5 ± 15.0	50.6 ± 14.1	0.569
Height (cm)	162.9 ± 9.7	166.8 ± 9.4	0.081
Weight (kg)	64.7 ± 12.2	68.8 ± 16.5	0.224
Sex (male/female)	14/24	21/17	0.167
Heart rate (bpm)	66 ± 12	72 ± 16	0.070
Systolic blood pressure (mmHg)	107 ± 20	107 ± 16	0.950
Diastolic blood pressure (mmHg)	65 ± 14	63 ± 13	0.644

Values are expressed as the mean ± SD or the number of patients

**Table 2:** Measured arterial diameter and depth from the skin to the radial artery using a cross-sectional ultrasound view at the puncture site

Parameter	Group C (*n* = 38)	Group E (*n* = 38)	*P* value
Transverse diameter of the artery (mm)	3.5 (2.6–4.2)	3.7 (2.8–4.2)	0.578
Longitudinal diameter of the artery (mm)	2.9 (2.4–3.6)	3.2 (2.8–3.6)	0.197
Arterial depth (mm)	3.2 (2.5–4.2)	3.9 (2.6–4.8)	0.296

Values are expressed as the median (interquartile)

There were no significant differences in the location of the puncture site (left/right; group C = 34/4 vs. group E = 35/3, *P* = 0.500) between the two groups (Table 3). All arterial cannulations were successful within three attempts. Comparing the success rates at the first attempt versus two and three attempts showed a significant difference between the groups (first/second and third; group C = 30/8 vs. group E = 36/2, *P* = 0.042). The median cannulation time for successful attempts in group E [seconds; 15 (11–19)] was not significantly different from that in group C [19 (13–24)] (*P* = 0.099).

**Table 3:** Puncture site, cannulation time, number of attempts, and complications during arterial cannulation

Parameter	Group C (*n* = 38)	Group E (*n* = 38)	*P* value
Puncture site (left/right)	34/4	35/3	0.500
Cannulation time (seconds)	19 (13–24)	15 (11–19)	0.099
Number of attempts (1/≥2)	30/8	36/2	0.042
Posterior wall puncture, n (%)	8	1	0.028
Haematoma, n (%)	8	1	0.028
Thrombosis, n (%)	0	0	–

Values are expressed as the median (interquartile) or the number of patients. Cannulation time is defined as the time taken from the initial skin puncture to the successful arterial catheter insertion; the number of attempts is defined as the number of new skin punctures required to puncture the radial artery. A posterior wall puncture is defined when the withdrawal of blood in the needle stopped after confirming the presence of occult blood while advancing the needle or when pulsatile blood appeared during the withdrawal of the needle; a haematoma is defined as a solid swelling of clotted blood around the artery confirmed by ultrasound. A thrombosis is defined as the local coagulation or clotting of blood in the artery confirmed by ultrasound

The incidences of posterior wall puncture (8% vs. 1%, *P* = 0.028) and haematoma formation (8% vs. 1%, *P* = 0.028) were significantly lower in group E than in group C, but no patients formed a thrombosis in either group.

## Discussion

The aim of this clinical study was to compare the success and complication rates during arterial cannulation between the electromagnetic guidance system and conventional ultrasound guidance. We found that the success rates on the first attempt were significantly improved, and the incidences of complications, such as posterior wall puncture and haematoma formation, were significantly decreased when arterial cannulation was performed under ultrasound guidance using the electromagnetic guidance system. While slightly favourable result in terms of cannulation time was also observed when the electromagnetic system was used, this finding was not statistically significant.

The electromagnetic guidance system was developed to overcome the drawbacks of conventional ultrasonography. With conventional ultrasonography, it is difficult to identify and predict the location of the tip of the needle during procedures [14]. The novel electromagnetic guidance system studied here made it easier to identify and predict the location of the tip of the needle because the location of the magnetized needle relative to the ultrasound probe is displayed on the screen. The predicted trajectory and actual position of the needle are also shown in the ultrasound view. In previous studies, the electromagnetic guidance system resulted in favourable outcomes when performing ultrasound-guided procedures using the phantom model [11–13]. However, the clinical advantages of the electromagnetic guidance system have not been previously validated. This study, as far as we know, is the first to investigate the effects of the electromagnetic guidance system in clinical practice.

The success rates on the first attempt were significantly better, and posterior wall punctures and haematomas occurred significantly less often when radial artery cannulation was performed under ultrasound guidance using the electromagnetic guidance system compared to conventional ultrasound guidance in this study. This result is likely because the electromagnetic guidance system enables both the identification of the location of the tip of the needle and prediction of the trajectory of the needle during the procedure. It can be difficult to identify the needle tip under conventional ultrasound guidance, which may result in the needle being inserted in a wrong location or advanced too deeply, causing a failure of the procedure or puncture in the posterior vessel wall and formation of a haematoma [4]. On the other hand, identifying the location of needle tip accurately using the electromagnetic guidance system prevents such outcomes. The risk of haematoma formation would be much higher in patients with uncontrolled hypertension or coagulation abnormalities, so the electromagnetic guidance system would lead to clinically favourable outcomes in these patients.

In this study, the short-axis view of the radial artery was obtained, and an out-of-plane approach was used to advance the needle during cannulation of the radial artery. It is impossible to monitor the movement of the needle in real time when an out-of-plane approach is used. On the other hand, the needle can be identified in real time when the procedure is performed using an in-plane approach. Thus, the electromagnetic guidance system can be more useful for procedures performed under ultrasound guidance using an out-of-plane approach compared to an in-plane approach. Thus, the results of this study could differ, depending on which approach is used. Applying the results of this study to procedures using different axes of the ultrasound view or approaches for needle advancement requires caution.

Among the many anatomical structures targeted for procedures under ultrasound guidance, the radial artery was the main focus of this study for several reasons. First, the radial artery is one of the most narrow structures in which cannulation is carried out with ultrasound guidance [3]. If the structure is narrow, targeting the needle is difficult, and a posterior wall puncture is more likely, even if ultrasound guidance is used. Second, the wall of the radial artery is exposed to higher pressures than that of a vein. There is a greater risk of complications such as haematoma formation made by a posterior wall puncture in the radial artery than in a vein. For successful cannulation of the radial artery without complications, a provider should advance the needle toward the centre of the radial artery and place the tip of the needle inside the radial artery precisely. Therefore, the radial artery was chosen as a target structure to validate the effectiveness and safety of the electromagnetic guidance system.

In this study, the cannulation time was slightly better in group E compared to group C, but the difference was not statistically significant. This finding could be due to the anatomical features of the radial artery, which include a cylindrical shape and shallow depth from the skin’s surface. Using an out-of-plane approach under ultrasound guidance, the vessel can be punctured without precisely guiding the location of the needle tip using the electromagnetic guidance system. Further, the relatively high pressure of the radial artery, compared to a vein, generates pulsatile blood flow in the catheter when punctured, making it easily visible so that the insertion of the needle tip into the arterial vessel can be confirmed. This reason may explain why the electromagnetic guidance system failed to show superior results in cannulation time compared with conventional ultrasound.

The indicator box, which shows the predicted location of the needle tip on the screen of the ultrasound device when the electromagnetic guidance system is used, could have influenced the results of this study. There were differences between the size of the indicator box and the radial artery, which was the cannulation target in this study. The indicator box is presented on the screen as a 5.0-mm square, but in this study, the diameters of the radial arteries were 3.5 mm (2.6–4.2 mm) in group C and 3.7 mm (2.8–4.2 mm) in group E, which are smaller than the indicator box. Therefore, it could be difficult for the provider to exactly locate the radial artery in the centre of the indicator box during the procedure. The results in the study may differ if the target vessel were larger than the indicator box.

This study has several limitations. First, the results of this study could be influenced by the skill and experience of the provider. A novel practitioner who is not used to the procedure might rely completely on the electromagnetic guidance and do not watch the actual needle tip during the procedure, which could incur some problematic situations. The anaesthesiologist in this study performed the procedures with and without the electromagnetic guidance system more than 50 times each. Therefore, applying the results of this study to practitioners with different skills and levels of experience compared with the anaesthesiologist in this study requires careful attention, especially for novel practitioners. Second, the provider could not be blinded because the activation of the electromagnetic guidance system was displayed on the screen. Third, we excluded patients who had vascular malformations or peripheral arterial occlusive disease in this study. Therefore, the effectiveness of the electromagnetic guidance on these patients is not known. Finally, a 20-G, 1.1 × 30-mm Angiocath Plus catheter was used for radial artery cannulation in this study. If a different kind of catheter is used, the results of this study could be different.

## Conclusions

In conclusion, the eZono 4000 electromagnetic guidance system improved the success rates on the first attempt and lowered the incidence of complications such as posterior wall puncture and haematoma formation during radial artery cannulation by an experienced practitioner.

## Data Availability

The datasets used and analysed during the current study are available from the corresponding author on reasonable request.

## Abbreviations

SD: Standard deviation
IQR: Interquartile range
